# Chick stem cells: Current progress and future prospects

**DOI:** 10.1016/j.scr.2013.09.005

**Published:** 2013-11

**Authors:** Sittipon Intarapat, Claudio D. Stern

**Affiliations:** Department of Cell and Developmental Biology and UCL Centre for Stem Cells and Regenerative Medicine, University College London, Gower Street, London WC1E 6BT, UK

## Abstract

Chick embryonic stem cells (cESCs) can be derived from cells obtained from stage X embryos (blastoderm stage); these have the ability to contribute to all somatic lineages in chimaeras, but not to the germ line. However, lines of stem cells that are able to contribute to the germ line can be established from chick primordial germ cells (cPGCs) and embryonic germ cells (cEGCs). This review provides information on avian stem cells, emphasizing different sources of cells and current methods for derivation and culture of pluripotent cells from chick embryos. We also review technologies for isolation and derivation of chicken germ cells and the production of transgenic birds.

## Highlights

• Chick embryonic stem cells (cESCs) can be derived from a variety of sources.
• cESCs can contribute to all somatic cell types but not to the germ line.
• germ cells can be isolated from early embryos, embryonic blood and gonads.
• germ cells can establish self-renewing lines and contribute to the germline.

## Graphical abstract

**Figure:**
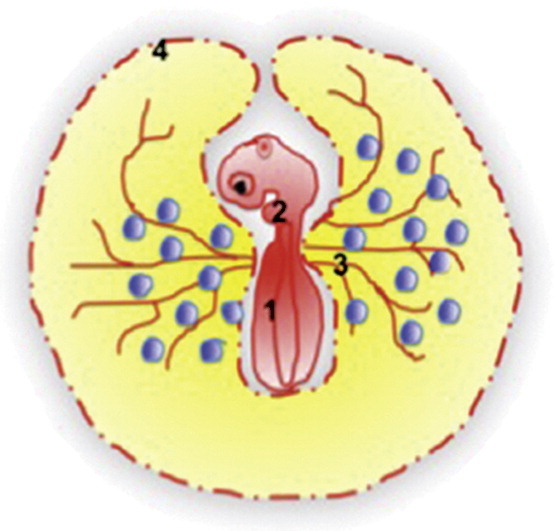

## Introduction

Avian embryos are a powerful model to study developmental and stem cell biology (Stern, 1996, 2004a, 2005). They offer several advantages as a model for studying stem cell biology including their convenient size and ease of obtaining eggs (Berg et al., 1999), their year-round availability and ease of access to the embryo for manipulations, which among other applications led it to be used as a favourite model for toxicity testing since very early days (Halldin, 2005; Halldin et al., 2005). To date, avians are the only non-mammalian group from which stable embryonic stem cell and germ cell lines have been established. Both cES and chick embryonic germ (cEG) cells are considered to be pluripotent (Petitte et al., 2004), but cES cells have been shown to be able to contribute only to somatic tissues and not to the germ line (Pain et al., 1996), while chick embryonic germ cells can contribute to the germ line (van de Lavoir et al., 2006a). However, surprisingly little attention has been given to the biology of avian stem cells, especially regarding similarities and differences between chick embryonic stem (cES) cells, germ cells, and stem cells obtained from other embryonic and adult tissues. Here we provide information on avian stem cells, emphasizing sources, methods for derivation and culture of pluripotent cells from chick embryos.

The avian embryo spends its first 20 h or so in utero; the shell is deposited as the egg descends down the maternal oviduct (for review see Stern, 2004b). During this time, cell division occurs in a meroblastic pattern (open cleavage planes, from the centre out to the yolk) to generate a disc. By the time the egg is laid, the blastodisc comprises 20,000–50,000 cells arranged mainly as a single-cell-thick layer (epiblast) underlain by islands of more yolky cells (hypoblast — extraembryonic endoderm of the future yolk sac stalk) (Stern, 2004b). The entire embryo will arise from the centre of the epiblast, but it retains a remarkable ability to regenerate. Fragments of blastodisc can regenerate the entire embryo and re-establish polarity (Bertocchini et al., 2004; Bertocchini and Stern, 2012; Spratt and Haas, 1960), suggesting plasticity of the embryo and perhaps pluripotency of the component cells. It is from these early (pre-primitive streak) stages of development that cell lines analogous to mammalian embryonic stem cells (ESCs) can be established from cells dissociated from the central epiblast; these cells can be perpetuated in culture, perhaps indefinitely (Etches et al., 1996, 1997; Pain et al., 1996).

The biology of germ cells in bird embryos is particularly interesting and unique. Primordial germ cells (PGCs) appear to arise at pre-primitive streak stages (see above) by ingression from the epiblast, joining the hypoblast cells below (Ginsburg, 1997; Ginsburg and Eyal-Giladi, 1986, 1987; Karagenc et al., 1996; Petitte et al., 1997). The hypoblast forms a continuous layer of cells that then moves to the most anterior part of the embryo, under the pre-amnion, carrying the PGCs to this region, known as the Germinal Crescent. One remarkable feature is that primordial germ cells use the embryonic blood vasculature as a vehicle to migrate out of the germinal crescent, until they eventually settle in the embryonic gonads (Fujimoto et al., 1976; Kuwana and Rogulska, 1999; Nakamura et al., 2007; Nieuwkoop and Sutasurya, 1979). Another unique characteristic of the gonads in female birds is that the right ovary regresses, and only the left ovary remains functional in the adult (Romanoff, 1960; Smith and Sinclair, 2001, 2004). However even male embryos have a greater number of PGCs in the left gonad (Intarapat and Stern, 2013).

To date, it has been possible to establish long-term, self-renewing cultures of cells from pre-primitive streak stage embryos and from germ cells isolated from the blood vasculature or from the gonad. A few cell lines have also successfully been established from later embryos and adult tissues. This review surveys our current knowledge about stem cells from these various sources and their main biological properties.

## Sources of chick stem cells

### Endogenous stem cells in the early embryo

**Figure 1:**
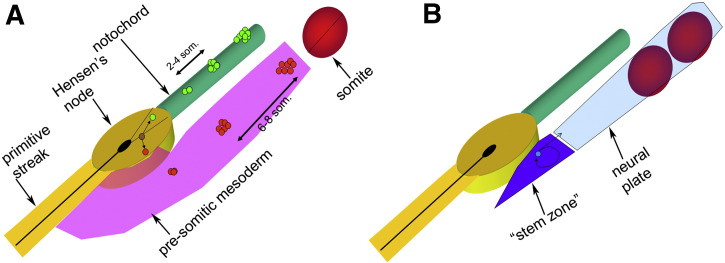
Endogenous stem cells in and around the primitive streak at early stages of chick development. A. From the full primitive streak stage (HH stage 4), the epiblast of Hensen's node contains a population of cells with properties suggesting that they are self-renewing, asymmetrically dividing stem cells. A single cell labelled in the median/anterior quadrant of the node generates clusters of labelled descendants in the notochord, about 2–4 somite-lengths apart. A single cell labelled a little more laterally generates similar clusters in the somites, 6–8 somites apart, suggesting that the cell cycle length is about twice the former (about 10 h) (Primmett et al., 1989). Occasionally, a cell labelled in the intermediate region can generate both types of clusters (Selleck and Stern, 1991, 1992). B. Next to the node, on each side of the epiblast, is a self-renewing region denominated “stem zone” which contains precursors for the caudal neural plate (which will give rise to the CNS, from hindbrain to tail) (Delfino-Machin et al., 2005). Eventually the node and stem zone seem to merge into a single domain containing mesendodermal precursors in the tail bud.

Endogenous stem cells were first discovered at early stages of chick embryo development by labelling a single cell in the organiser, Hensen's node, with fluorescent lineage tracers and following its descendants over time (Selleck and Stern, 1991, 1992; Stern et al., 1992). As the axis is laid down, the labelled cell deposits regularly spaced clusters of descendants along the axis (within the notochord and/or somite mesoderm), often with a single marked cell remaining at the site of labelling. Each cluster contains twice as many cells and displays about half the fluorescence intensity than the next cluster, consistent with the idea of resident cells that divide asymmetrically, one daughter remaining in place and the other contributing to emerging structures such as notochord and somites (Selleck and Stern, 1991, 1992; Stern et al., 1992) (Fig. 1A). The gradual ingression of cells from Hensen's node into the pre-somitic mesoderm, at a constant position in the cell cycle, has been related to somite formation, underlying the cell cycle model for somite formation (Collier et al., 2000; Palmeirim et al., 2008; Primmett et al., 1989; Stern et al., 2006). Since then, results consistent with this have been obtained in the mouse (Nicolas et al., 1996; Wilson et al., 2009).

A separate region containing resident stem-cell-like cells has also been shown to exist in the epiblast just lateral to Hensen's node by Storey and colleagues (Akai et al., 2005; Delfino-Machin et al., 2005; Diez del Corral et al., 2003; Wilson et al., 2009) (Fig. 1B). This small region contributes cells to the caudally-elongating neural tube that will form the central nervous system from the hindbrain to the tail. Proliferation of the progenitor cells in the stem zone is maintained by FGF and Notch signalling and opposed by retinoids, causing cells to stop dividing, acquire Pax6 expression and differentiate into neurons (Akai et al., 2005; Delfino-Machin et al., 2005; Diez del Corral et al., 2003). Presumably both regions containing stem cells persist into the tail bud of later stage embryos, within a region where ectoderm and mesoderm merge into a solid mass and from which cells can contribute both to mesoderm (notochord and somite) and to the ventral neural tube. A striking demonstration of the self-renewing character of this region was provided by serial transplantation between GFP-transgenic and wild-type chick embryos: the grafted transgenic tail bud contributed extensively to the axis over at least two generations of host embryos (McGrew et al., 2008). Although in this latter case the phenomenon of self-renewal has not yet been studied at the single cell level (as in the node, see above), the results are consistent with this interpretation.

### Chick embryonic stem cells (cESCs)

**Figure 2:**
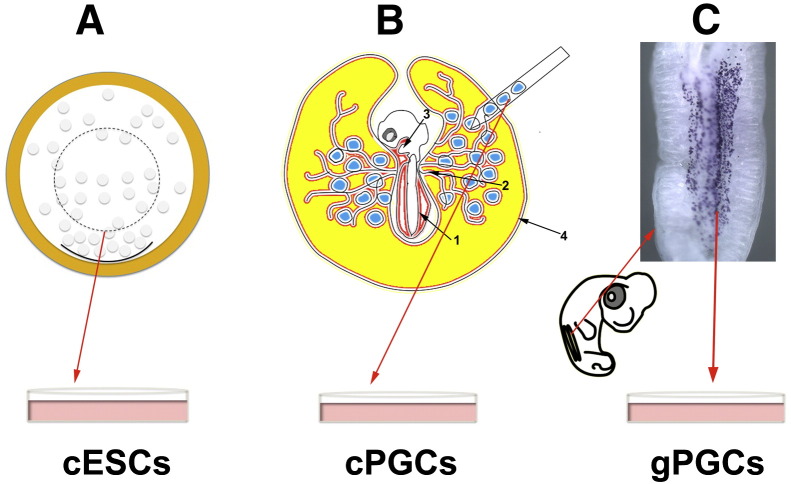
Sources of pluripotent stem cells from chick embryos at different stages of development. A. Chick embryonic stem cells (cESCs) can be derived from blastodermal cells at stage X (EG&K) (A). B. Circulating or blood derived-primordial germ cells (cPGCs) and chick embryonic germ cells (cEGCs) can be isolated from the dorsal aorta (1), vitelline artery (2), embryonic heart (3) or sinus terminalis (4) of stage 14 (H&H) embryos. At this stage, cPGCs use blood vessels as a route to migrate from the circulatory system to the future gonads. C. Gonadal primordial germ cells (gonocytes, gPGCs) and chick embryonic germ cells can be obtained from stage 25–28 (H&H) embryos. At this stage future gonads can be seen as bilateral ridges called gonadal or genital ridges where gPGCs have reached and settled inside to develop into functional gametes. GR = gonadal ridges; blue dots represent gPGCs.

**Figure 3:**
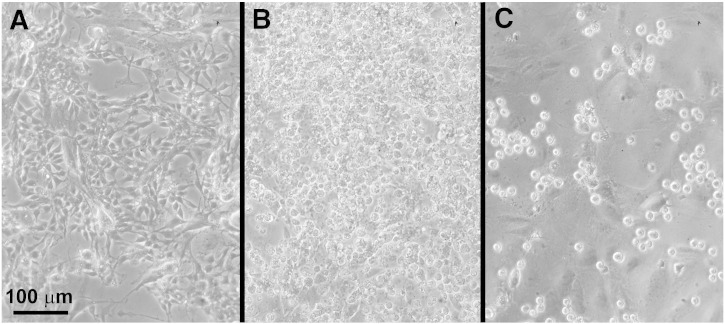
Morphology of chick embryonic stem cells (cESCs), circulating-primordial germ cells (cPGCs) and gonadal-primordial germ cells (gPGCs). A. The established cESC line 9N2 (Pain et al., 1996) has typical characteristics of undifferentiated embryonic stem cells with prominent large nucleus and relatively little cytoplasm. B. The established cPGC line NuGFP-02, isolated from embryonic blood (van de Lavoir et al., 2006a), can easily be distinguished from BRL feeder cells by having large cells with large nuclei and refractive granules in the cytoplasm. C. The established gPGC line 527, isolated from embryonic gonads (van de Lavoir et al., 2006a). Scale bar = 100 μm.

Most current work in the stem cell field is done using mammalian cells in vitro, particularly mouse and human; to date, the only non-mammalian system from which pluripotent embryonic stem cell lines can be established is the avian system, especially the chick. In chick, pluripotent cells have been isolated from several sources (summarised in Tables 1 and 2) and various sources of chick pluripotent stem cells from different stages of development and morphology of cESC, cPGCs and cEGCs are shown in Figs. 2–3.

#### Isolation, culture and characterization of cESCs in vitro

As in mouse, cell lines can be established from cells obtained from very early chick embryos, prior to gastrulation. However, unlike murine ESCs, these chick ESCs have been shown to be able to contribute only to somatic lineages but not the germline (Lavial and Pain, 2010). Freshly isolated chick blastodermal cells retrieved from the area pellucida of stage X (Eyal-Giladi and Kochav, 1976; EG&K) embryos can contribute to all somatic tissues as well as the germline after injection into the subgerminal cavity of stage X (EG&K) recipient embryos (Carsience et al., 1993; Kagami et al., 1995; Kino et al., 1997; Petitte et al., 1990), but germline potency seems to be lost rapidly in culture. Thus, cESCs are more similar to murine epiblast stem cells (EpiSCs) (Lavial and Pain, 2010) and to human embryonic stem cells (hESCs) (Thomson et al., 1998) than to mESCs. This could be due to the fact that stage X is developmentally more advanced than the mouse ICM, from which mESCs are derived; it is possible that primordial germ cells have already left the epiblast by stage X, or that their lineage has already separated from somatic fates. That the ability of this cell population to contribute to the germline is lost upon culture suggests that early PGCs do not survive the culture conditions used in these studies, while the remaining epiblast-derived cells are already restricted to somatic lineages.

Many germline-associated genes in chick have been reported including *Deadend* (Aramaki et al., 2007), *Dazl* (Rengaraj et al., 2010), *Piwi* (Kim et al., 2012) and the chick *vasa* homologue (*Cvh*), which was the first of these genes cloned in chick (Tsunekawa et al., 2000). *Cvh* expression has been detected from stage X (EG&K) until adult stages (Tsunekawa et al., 2000); in the latter, expression is restricted to functional male and female gametes (Tsunekawa et al., 2000).

The limited ability of cESCs to contribute to the germline lineage could be explained by early germline determination and the reduction of germline potency in vitro (Lavial et al., 2009). *Cvh* might be a key gene for germ cell specification and determination in chick that enables cPGCs to enter the germline. Indeed, cESCs transfected with *Cvh* plasmid and cultured in differentiation medium in vitro adopt a germ cell fate (Lavial et al., 2009). This suggested that *Cvh* may be sufficient to confer cESCs with the ability to contribute to the germline in vivo by colonising the embryonic gonads, expressing specific germline and meiotic markers (Lavial et al., 2009).

**Figure 4:**
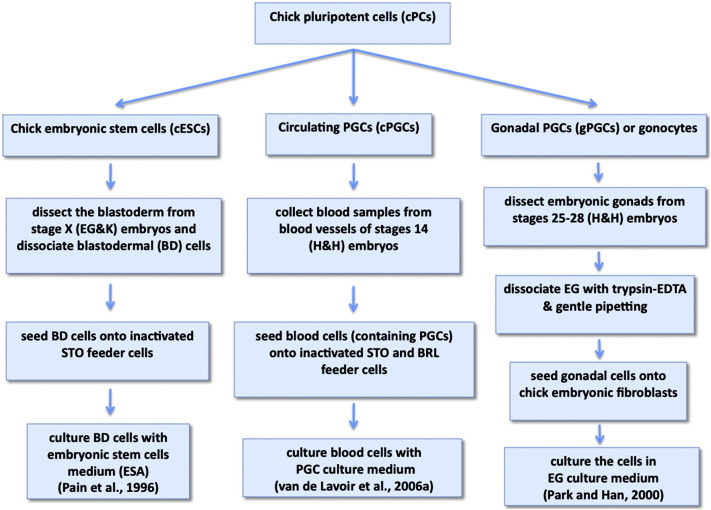
Methods for isolation and derivation of chick pluripotent cells. Table summarising the main features of current methods for deriving cell lines from different sources.

Chick ESCs were first isolated from stage X blastodermal cells by culturing them on inactivated STO feeder cells in embryonic stem cell medium (ESA) containing growth factors and cytokines including bFGF, IGF-1, mSCF, IL-6, IL-11, CNTF, OSM and LIF (Aubel and Pain, 2013; Pain et al., 1996). Like mESCs, cESCs can be maintained in an undifferentiated state in the presence of LIF (Horiuchi et al., 2004, 2006). Current methods for isolating cESCs are summarised in Fig. 4.

Several characteristics have been shown to be shared between cESCs and their mESC counterparts. First, alkaline phosphatase (AP) activity is exhibited by cESCs (Pain et al., 1996; van de Lavoir and Mather-Love, 2006; van de Lavoir et al., 2006b). Several immunological markers are also expressed, including stage-specific embryonic antigens (SSEA) SSEA1, SSEA3 and SSEA4 (Knowles et al., 1978; Pain et al., 1996; Shevinsky et al., 1982; Solter and Knowles, 1978). Expression of chick homologues of Oct3/4 (*cPouV*) and *cNanog* has also been reported in cESCs (Lavial et al., 2007).

Being able to differentiate is one of the characteristics of ESCs; cESCs can generate nerve cells, haematopoietic cells and muscle cells and can also form embryoid bodies when plated onto low adherence plates in medium without LIF (Pain et al., 1996). Removal of LIF from the culture medium causes loss of SSEA1 (Pain et al., 1996), *cPouV* and *cNanog* expression (Lavial et al., 2007).

### Chick somatic and adult stem cells

#### Neural stem cells

Neural stem cells (NSCs) are multipotent cells with the ability to self-renew and to differentiate into various cell types of the nervous system such as neurons and glia (Alvarez-Buylla et al., 2001; Temple, 2001). In adult birds, it was reported long ago that precursor cells located in the ventricular zone of the forebrain give rise to new neurons (Goldman and Nottebohm, 1983; Nottebohm, 1985; Paton and Nottebohm, 1984). Radial glia in contact with the ventricle appear to act as stem cells, which give rise to new neuroblasts (Alvarez-Buylla et al., 1990, 1998).

In chick, NSCs have not been found to be present in substantial numbers in the central nervous system until after embryonic day 5; SOX9 appears to be crucial for the formation and maintenance of NSCs (Scott et al., 2010). Cells with NSC properties can also be isolated from adult CNS; these cells are able to form clusters of cells known as neurospheres in long term in vitro culture. The majority of in vitro experiments use neurospheres as an assay for the presence of NSCs (Reynolds and Rietze, 2005). Neurospheres can be generated for example by culturing chick spinal cord with neurobasal A medium supplemented with EGF and FGF-2 (Whalley et al., 2009). However, little is known about the molecular mechanisms that regulate the maintenance, proliferation and differentiation of NSCs in the adult chick CNS.

Recently, the lateral ventricle of chick embryonic brain was used to assess the behaviour of human neural stem cell (hNSC) properties in vivo (Kharazi et al., 2013). hNSCs successfully engrafted into chick embryonic brain, introducing a new model for studying human stem cells in the nervous system (Kharazi et al., 2013).

#### Mesenchymal stem cells

Mesenchymal stem cells (MSCs) (also known as mesenchymal progenitors, stromal stem cells or multipotent mesenchymal stromal cells) were first described from bone marrow cultures as cells having fibroblast-like shape, colony forming ability and adherent to plastic (Dominici et al., 2006; Friedenstein et al., 1982, 1987). They have been shown to be able to self-renew and to differentiate into a variety of cell types (Bruder and Caplan, 1990; Bruder et al., 1994; Jaiswal et al., 1997; Javazon et al., 2004; Lee et al., 2010; Owen and Friedenstein, 1988; Pittenger et al., 1999; Prockop, 2009; Reese et al., 1999; Saito et al., 1995; Wakitani et al., 1994, 1995; Yoo et al., 1998; Yoshimura et al., 2006; Young et al., 1998), which led to the proposal of the “mesengenic progress”, defined as the ability of bone marrow derived MSCs to give rise to mesenchymal lineages including bone, cartilage, muscle, marrow, tendon, ligament and connective tissue (Caplan, 1994). Several makers have been proposed to characterize MSCs including CD73/SH3 (Barry et al., 2001; Chartoff et al., 2011), CD90/Thy1 (Giuliani et al., 2011) and CD105/SH2/Endoglin (Barry et al., 1999; Chartoff et al., 2011; Kern et al., 2006; Ninagawa et al., 2011; Trivedi and Hematti, 2008). Other commonly used indicators include absence of expression of haematopoietic (CD34 and CD45) and endothelial (CD31) markers, co-expression of CD105, CD90/Thy-1, CD73, CD44/HCAM, CD166/ALCAM, CD29 (Pittenger et al., 1999) and CD146/MSCA-1 (Sorrentino et al., 2008). MSCs can also be isolated from various somatic organs such as lung (Chow et al., 2011; Gong et al., 2012), heart (Anzalone et al., 2013; Vasa et al., 2001), umbilical cord (Lee et al., 2004; Tsagias et al., 2011; Weiss and Troyer, 2006; Zhang et al., 2011) and bone marrow (Kastrinaki et al., 2008; Romanov et al., 2005; L. Wang et al., 2009). Because of their great multipotentiality, there has been great hope that MSCs can be used for regenerative medicine. This has been explored for diseases of the CNS (stroke, Multiple Sclerosis, Amyotrophic Lateral Sclerosis) (Estes et al., 2006; Mahmood et al., 2003; Riordan et al., 2009), the bone marrow (GvHD) (Bernardo et al., 2011; Le Blanc et al., 2008), the heart (chronic/AMI) (Pittenger and Martin, 2004; Toma et al., 2002), the lung (asthma, Cystic Fibrosis) (Bonfield and Caplan, 2010; Bonfield et al., 2010; Solchaga et al., 2005), muscle (Muscular Dystrophy) (Bosch et al., 2000; Lee et al., 2000), pancreas (diabetes) (Vija et al., 2009) and for alleviation of spinal cord injury (Chopp and Li, 2002; Neuhuber et al., 2005).

Like their mammalian counterparts, chick MSCs have been isolated from different organs including liver (Mu et al., 2013), lung (Khatri et al., 2010), bone marrow (Bai et al., 2013a; Khatri et al., 2009, 2010; Khatri and Sharma, 2009) and umbilical cord Wharton's jelly (Bai et al., 2013b). Chick MSCs from different organs can be differentiated into osteogenic and adipogenic lineages. However, whether MSCs from different tissue sources are actually the same cell type, and whether they have equivalent potency, is unknown. To date, it has been reported that umbilical cord-derived cMSCs can differentiate into cardiomyogenic cells (Bai et al., 2013b), and that bone marrow-derived cMSCs can differentiate into endothelial (Bai et al., 2013a) and chondrogenic cells (Khatri et al., 2009).

#### Muscle stem cells

The growth of adult skeletal muscle depends on the proliferation and differentiation of muscle progenitor cells derived from specialised muscle stem cells, the satellite cells (Brack and Rando, 2012; Buckingham, 2006; Montarras et al., 2005; Relaix et al., 2005). In both chick and mouse, satellite cells and muscle progenitors reside under the basal lamina (Cossu and Biressi, 2005; Halevy et al., 2006; Yablonka-Reuveni, 1995). Muscle progenitors have been shown to arise from the dermomyotome until late during embryogenesis and eventually become located under the basal lamina of muscles (Picard and Marcelle, 2013). It was suggested that the cellular strategies driving muscle growth in embryonic and fetal stages have been conserved during the evolution of amniotes (reptiles, birds and mammals) (Picard and Marcelle, 2013). Two distinct populations of muscle progenitor cells appear to coexist throughout amniote development; a common feature is that Pax7 expressing cells co-exist with a major fast-cycling population and Myf5 expressing cells (Picard and Marcelle, 2013), consistent with a model (Rudnicki et al., 2008) of muscle homeostasis during development.

In the chick, satellite cells have been isolated from Beijing fatty chicken (Bai et al., 2012). These cells were characterized molecularly using MyoD, Pax7 and desmin as myogenic markers and were found to differentiate into osteocytes and adipocytes after exposure to bone morphogenetic (BMPs) and adipogenic factors, respectively (Bai et al., 2012).

#### Amniotic stem cells

Like mammals, avian embryos contain extra-embryonic membranes including allantois, yolk sac, chorion and amnion. The cavity enclosed by the amnion contains fluid and a population of stem cells that expresses Oct4 (Prusa et al., 2003). Cells isolated from the mammalian amniotic cavity can generate clonal cell lines and can differentiate into primary germ cell lineages (De Coppi et al., 2007). These cells are known as amniotic fluid stem cells (AFSCs) and express c-kit surface antigen (CD117), the receptor of stem cell factor (Zsebo et al., 1990). Because of the ease of isolation using relatively non-invasive methods in humans, AFSCs are considered a promising source of stem cells for regenerative medical applications (Bajada et al., 2008).

Although the vast majority of studies on AFSCs have been done in mammals (Carraro et al., 2008; De Coppi et al., 2007; Elwan and Sakuragawa, 1997; Fuchs et al., 2004; Perin et al., 2007; Steigman et al., 2009), stem cells have also recently been isolated from chick amnion (Gao et al., 2012). Chick amniotic epithelial cells (cAECs) isolated from 6 day old chick embryos not only express CK19 (an epithelial cell marker) but also the pluripotency-associated genes *Oct4*, *Nanog* and *Sox2* (Gao et al., 2012). Moreover, they have been successfully induced to differentiate into pancreatic islet-like cells, osteoblasts, adipocytes and neural-like cells (Gao et al., 2012), suggesting that cAECs are multipotent.

#### Germline stem cells

In chick, germline stem cells have been studied mainly in connection with the production of transgenic animals. Several techniques for transgenesis using male germline stem cells, also called spermatogonial stem cells (SSCs), have been described. These include testis-mediated gene transfer (Li et al., 2008), transplantation of transfected SSCs (Li et al., 2008), electrotransfection (Yu et al., 2010), allogeneic transplantation (Yu et al., 2010), lentiviral infection (Liu et al., 2010) and busulfan treatment (Tagirov and Golovan, 2012).

Since the first report of isolation of chick germline stem cells from testes (Jung et al., 2007), a testis-mediated system has been devised for transgenic technology (Han, 2009; Lee et al., 2006). This facilitates the isolation and derivation of pluripotent cell lines from adult stages. Both PGCs and SSCs can be differentiated into adipocytes, neuron-like cells and osteoblasts in vitro (B. Li et al., 2010; B.C. Li et al., 2010) and both express similar gene markers (Jung et al., 2005, 2007).

Although there are many publications about male germline stem cells in chick, there is as yet no evidence for an equivalent female germline stem cell. However, chick ovarian cells have been cultured for toxicology (Liu et al., 2006; Xie and Zhang, 2004), endocrinology (Liu et al., 2005; Sirotkin and Grossmann, 2007; Velazquez et al., 2006) and cancer-related (Giles et al., 2006) studies.

#### Avian induced pluripotent stem cells (iPSCs)

Direct reprogramming has been established recently for converting differentiated somatic cells into pluripotent ES-like cells. This method is useful for medical applications and avoids ethical issues associated with the use of human eggs or embryonic tissues. It has been hypothesised that fusion of somatic cells with ES cells can generate ES-like cells by inducing the pluripotent state and de-differentiation (Yamanaka, 2008). Key factors able to induce mouse embryonic and human fibroblasts into iPSCs have been discovered; these factors can maintain pluripotency both in vitro and in vivo (Takahashi et al., 2007a, 2007b; Takahashi and Yamanaka, 2006). The original method used for testing pluripotency-inducing factors involved retrovirus-mediated transfection; when four transcription factors (Oct-4, Sox2, c-Myc and Klf4) were transfected into mouse fibroblasts, a pluripotent stem cell state was induced. Cells generated this way are known as induced pluripotent stem cells (iPSCs) (Takahashi and Yamanaka, 2006), and these transcription factors are now often referred to as “Yamanaka factors”. iPSCs generated by this method are similar to mESCs including having an ES cell-like morphology, proliferation and the ability to form teratomas (Takahashi and Yamanaka, 2006).

iPSCs have been successfully generated from several mammalian species including human (Imamura et al., 2012; Okita et al., 2013; Okita and Yamanaka, 2010; Qu et al., 2012; Sommer et al., 2012; Takahashi et al., 2007b; Yu et al., 2009, 2010; Zhou et al., 2012), mouse (Imamura et al., 2012; Okita et al., 2008; Takahashi et al., 2007a; Takahashi and Yamanaka, 2006), common marmoset (Imamura et al., 2012; Wiedemann et al., 2012), monkey (Wunderlich et al., 2012; Zhong et al., 2011) and pig (Liu et al., 2012; Rodriguez et al., 2012; West et al., 2010). The combination of factors used differs a little among different groups and for different species; for example, Thompson's group uses Oct-4, Sox2, Nanog and Lin28 transfected into human foreskin fibroblasts with lentiviral vectors (Yu et al., 2007). However, iPSCs can be generated successfully in various mammalian species using human reprogramming factors, suggesting that the reprogramming mechanisms are conserved in mammals.

The first non-mammalian iPSCs were recently generated from quail using embryonic fibroblasts (QEFs) transfected with lentiviral vectors containing human *POU5F1*, *NANOG*, *SOX2*, *LIN28*, *KLF4*, and *C-MYC* (Lu et al., 2012). This is a larger number of reprogramming factors than routinely used for mammalian cells (selected by combining the Yamanaka and Thompson sets of factors). It seems likely that a smaller subset should also be successful, but this has not yet been explored systematically. Nevertheless, this pioneering study reveals that reprogramming somatic cells by a few transcription factors is not a unique property of mammalian cells, and, since human proteins work on quail cells, that the molecular mechanisms of reprogramming may be largely conserved beyond mammals. Like their mammalian counterparts, quail iPSCs exhibit pluripotent stem cell characteristics including differentiation into derivatives of the primary germ layers, neural differentiation, embryoid body formation and importantly the ability to produce germ line chimaeras (Lu et al., 2012). It should therefore be possible to generate chick iPSCs.

## Applications and technologies related to chick stem cells

### Isolation and derivation of chick embryonic stem cells

Methods for isolation and derivation of cESC lines were first reported by Pain et al. (1996). They used cells derived from the chick blastoderm before gastrulation (stages IX–XI), dissociated mechanically in ESA medium, to establish lines. In culture, the cells expressed ECMA-7 and SSEA-1 and were able to differentiate into somatic tissues in vitro and to contribute to the germline in vivo (Etches et al., 1997). For longer-term culture, the cells were grown on a feeder layer of inactivated STO cells (a mouse embryonic fibroblast cell line), supplemented with a cocktail of growth factors (see above). Under these conditions, cESCs exhibited similar characteristics to mESCs (see Table 1). Since then, another method was designed to improve the ease of isolation and establishment of the cell lines and the efficiency of production of somatic chimaeras from cultured cells (van de Lavoir and Mather-Love, 2006; van de Lavoir et al., 2006b). This method also uses cells dissociated from the area pellucida rather than from the whole embryo (which contains a large extraembryonic area opaca region) used for the earlier studies. To maintain undifferentiated cESCs in culture for long periods, Leukaemia Inhibitory Factor (LIF) appears to be important (Nichols et al., 1994; Smith and Hooper, 1987). Both natural LIF (secreted by BRL cells) (van de Lavoir and Mather-Love, 2006; van de Lavoir et al., 2006b) and synthetic LIF (Pain et al., 1996) have been used successfully for maintaining cESCs in an undifferentiated state. van de Lavoir's and Pain's methods both relied on murine LIF (mLIF), but other groups have sought to use conspecific, recombinant chicken LIF (cLIF) which was reported to promote alkaline phosphatase and EMA-1 expression (Horiuchi et al., 2004, 2006). Other recent methods use enzymatic dissociation to obtain the cells (Zhang et al., 2013), combined with the use of chick embryonic fibroblasts (CEFs) as feeder cells instead of STO cells, along with culture in medium without added growth factors. cESCs grown this way express alkaline phosphatase and SSEA-1 and can contribute to somatic tissues in chimaeras.

Methods for culture of other avian somatic and adult stem cells are summarised in Table 2. A wide variety of isolation conditions, media and feeder layers have been used by different groups. However no rigorous, systematic comparison of these conditions has been undertaken for cells from any of these different tissue sources and it is therefore impossible to determine which of these conditions are important for specific properties of the stem cell lines, or whether these vary according to the originating tissue type.

### Isolation and derivation of chick germ cell lines

Several attempts have been made to isolate chick germ cells and to establish cell lines from them. It was first demonstrated that chick PGCs can be cultured from pre-primitive-streak stage embryos (Karagenc et al., 1996); factors secreted by STO cells were found to enhance their maintenance (Karagenc and Petitte, 2000). PGCs can also be obtained from the germinal crescent of primitive streak stage embryos, which can be successfully transfected by retroviruses; recipient embryos injected with such transfected PGCs grew to sexual maturity and produced offspring containing the foreign DNA (Vick et al., 1993).

Chick PGCs have some unique characteristics that distinguish them from their mammalian counterparts, such as their use of the blood circulation as a migratory route to the embryonic gonad. This allows PGCs to be isolated from embryonic blood. This was achieved relatively recently (van de Lavoir et al., 2006a). Blood-derived PG cells remained undifferentiated after prolonged culture in the presence of LIF, SCF and bFGF (van de Lavoir et al., 2006a); cells established by this method exhibit good germline transmission after injection into stage 13–15 embryos, but do not contribute to somatic tissues, suggesting that they are committed to a germline fate. Genetically modified PGCs have been created from these cells (van de Lavoir et al., 2006a), offering a method for production of transgenic lines of birds.

Several attempts have also been made to isolate gonadal-derived germ cells from later embryonic stages (Chang et al., 1995, 1997; Ha et al., 2002; Park and Han, 2000; Park et al., 2003; Shiue et al., 2009; Suraeva et al., 2008; J. Wang et al., 2009; Wu et al., 2010) (see Table 3). However, very few germline chimaeras were obtained after injecting cultured gonad-derived germ cells into recipient embryos (Chang et al., 1995, 1997; Ha et al., 2002; Park et al., 2003). IGF and IL-11 were reported to be essential for gonadal PGCs to maintain germline potency and colony formation (Chang et al., 1995; Park and Han, 2000). Although no systematic comparison has yet been undertaken, these findings suggest that blood-derived and gonadal-derived germ cells may differ in their ability to be cultured for long periods and in their capacity to establish germline chimaeras. A method for isolating and deriving chick embryonic germ cells is summarised in Fig. 4.

### Production of transgenic birds

Although retroviral vectors have been used to transduce exogenous genes into early chick blastoderms and somatic stem cells, and constructs including LacZ can be transfected into cells of the blastodisc to produce chick chimaeras (Inada et al., 1997; Naito et al., 1991, 1994), the transgene has not yet been shown to be transmitted through the germline (Bosselman et al., 1989). In contrast, lentiviral vectors have been used with considerable success to generate several transgenic lines of birds (both chick and quail) (Bosselman et al., 1989; Fraser et al., 1993; Hunter et al., 2005; Lillico et al., 2005; McGrew et al., 2004; Poynter et al., 2009a, 2009b, 2009c; Poynter and Lansford, 2008; Sang, 2004; Sato and Lansford, 2013; Sato et al., 2010; Seidl et al., 2013). The method is now almost routine and several laboratories are now establishing transgenic lines of birds for example expressing GFP (cytoplasmic or membrane-localized) ubiquitously or driven by tissue-specific promoters, and other cell lines such as reporters for various signalling pathways for research applications. A particularly promising technique for transgenesis was recently described, using the Tol2 and piggyBac transposons, allowing the introduction of large inserts (Macdonald et al., 2012).

Despite this success for transgenic bird construction, lentiviral transduction does not easily allow targeted mutagenesis at a selected gene locus because the transgene inserts randomly at multiple sites. Although carefully designed breeding can separate lines and purify those with a single integration, it is still not possible to target specific gene loci using this method. For this, a cell-based method using homologous recombination in ESC or PGC would be desirable. This has not yet been achieved, despite the fact that homologous recombination is possible, and even relatively straightforward, in avian cells (Acloque et al., 2001, 2004; Ishiai et al., 2012; Takata et al., 2006, 2009). A major hurdle remains the availability of cell lines, susceptible to homologous recombination (and which can be maintained in culture so that selection can be used to isolate lines that have undergone successful recombination, as in mouse), and able to contribute to the germ line.

Blastoderm-derived cells were first used for creating chimaeric chickens after transplanting these cells to the central zone (Marzullo, 1970) or the subgerminal cavity of early embryos (Pain et al., 1996; van de Lavoir and Mather-Love, 2006; van de Lavoir et al., 2006b; Zhang et al., 2013). Some success has been obtained with early blastodermal cells containing PGCs from stage X embryos, leading to the production of both somatic and germline chimaeras (Carsience et al., 1993; Etches et al., 1996; Kagami et al., 1995; Petitte et al., 1990; Thoraval et al., 1994). Both fresh and cryopreserved blastodermal cells were used for chimaera production, although fresh cells were more efficient (Etches et al., 1996; Kino et al., 1997).

Since long-term cultured cESC lines do not seem to be able readily to contribute to the germ line, and since lines of PGCs are difficult to establish, a method for reprogramming cESCs to acquire germline competence would be desirable. A recent study demonstrated that cESCs transfected with *Cvh* in an expression plasmid can colonise the embryonic gonad and express the germ cells' marker DAZL (Lavial et al., 2009). One study claims to have produced transgenic chicks using transdifferentiated chicken bone marrow cells transplanted into the testes (Heo et al., 2011).

Germ cell-based methods for transgenesis in the chick were described long ago, in a study using germinal crescent-derived PGCs along with a replication-deficient retroviral vector (Vick et al., 1993). Lentiviral vectors can also be used to introduce transgenes into gonadal-PGCs (Shin et al., 2008). Blood-derived PGCs have also been used successfully to generate transgenic chicks, using electroporation for gene transfer (van de Lavoir et al., 2006a). Methods that rely on PGCs for creating transgenic birds have been called “embryo-mediated system” (Han, 2009). A “testis-mediated system” has also been described (Lee et al., 2006); this method is said to be advantageous because it eliminates the need for PGC retrieval and reduces the time for the test cross (Han, 2009). However, a comparison between embryo-mediated and testis-mediated systems for obtaining high yields and efficient production of transgenic chicks has yet to be undertaken.

Thus, although cell-based methods do hold promise for targeted mutagenesis in the chick, this has yet to be successfully achieved. At present, lentiviral vector-mediated transgenesis appears to be the most efficient method for producing transgenic bird lines, but this does not allow targeted mutagenesis of endogenous loci.

## Conclusions

Chick stem cells can be obtained from embryos and maintained in culture. They can be derived from different sources at various stages of embryonic development. They have been demonstrated to be pluripotent because they can form embryoid bodies, differentiate into cell types from all three embryonic germ layers and contribute to somatic and germline lineages in chimaeras. They are therefore comparable to mammalian stem cells, offering a model for studying stem cell biology as well as being a tool for many applications.

## Conflict of interest

The authors declare no conflict of interest.

## Figures and Tables

**Table 1:** Pluripotent stem cell types from avian embryos.

Cell type	Source	Confirmation methods	References
ESCs	Stage X (EG&K)	EB formation, in vitro differentiation, somatic chimaeras	1, 2, 3
PGCs	Stages 14–17 (H&H)	Germline chimaeras	4
EGCs	Stage 28 (H&H)	EB formation, in vitro differentiation, somatic chimaeras	5
GSCs/SSCs	Juvenile 6 wk old and adult (24-wk old) male roosters	EB formation, in vitro differentiation	6, 7
iPSCs	Quail embryonic fibroblasts (embryonic day 11)	EB formation, in vitro differentiation, germline chimaera	8

Abbreviations: ESCs = embryonic stem cells, PGCs = primordial germ cells, EGCs = embryonic germ cells, GSCs = germline stem cells, SSCs = spermatogonial stem cells, iPSCs = induced pluripotent stem cells; EG&K = stage (Eyal-Giladi and Kochav, 1976), H&H = stage (Hamburger and Hamilton, 1951). EB = embryoid body. References: 1. Pain et al. (1996). 2. Boast and Stern (2012); van de Lavoir et al. (2006b). 3. Petitte et al. (2004). 4. van de Lavoir et al. (2006a). 5. Park and Han (2000). 6. Lee et al. (2006). 7. Jung et al. (2007). 8. Lu et al. (2012).

**Table 2:** Avian somatic and adult stem cells.

Cell type	Culture conditions	Differentiation	Culture duration	References
Neural stem cells	Neurobasal A + EGF + FGF2 heparin	Neurosphere	7–14 days	1
Mesenchymal stem cells				
Liver-MSCs	DMEM/F12 + FBS + bFGF	Neuronal/osteoblast cells	7–8 days	2
Lung-MSCs	DMEM + FBS + HEPES	Adipogenic/osteogenic cells	21 days	3
Bone marrow-MSCs	DMEM + FBS	Adipogenic/osteogenic/chondrogenic cells	14–21 days	4
	L-DMEM + FBS	Adipogenic/osteogenic/endothelial cells	6–20 days	
Umbilical cord-MSCs	L-DMEM + FBS	Adipogenic/osteogenic/cardiomyogenic cells	7–21 days	5
Muscle stem cells	DMEM/F12 + FBS + bFGF	Myogenic/osteogenic/adipogenic	6 days	6
Amniotic stem cells	DMEM/F12 + FBS	Neuronal/osteogenic/adipogenic/pancreatic like cells	7–14 days	7
Germline stem cells	Modified-DMEM + FBS + CS + HEPES + LIF + FGF2 + IGF1	EB formation/three germ layers formation/germline chimaera	21 days	8
Induced pluripotent stem cells (quail)	H-DMEM + FBS	EB formation/three germ layers formation/chimaera	7–39 days	9

References: 1. Whalley et al. (2009); Reynolds and Rietze (2005). 2. Mu et al. (2013). 3. Khatri et al. (2010). 4. Khatri et al. (2009); Bai et al. (2012). 5. Bai et al.(2013a). 6. Bai et al.(2013b). 7. Gao et al. (2012). 8. Jung et al. (2007); Lee et al. (2006). 9. Lu et al. (2012).

**Table 3:** Comparison of methods for isolation and derivation of blood-derived and gonadal-derived PGCs.

Cells	Feeder layer	Sera/growth factors/cytokines	References
cPGCs	Irradiated BRL	FBS, CS/bFGF, SCF/secreted LIF from BRL	van de Lavoir et al. (2006a)
		FBS, CS/–/–	Yamamoto et al. (2007)
		FBS, CS/bFGF, SCF, hLIF	Choi et al. (2010)
	Irradiated STO	FBS, CS/FGF, SCF/secreted LIF from BRL	Macdonald et al. (2010)
gPGCs	CEF	FBS, CS/bFGF, SCF, IGF-I/mLIF, IL-11	Park and Han (2000)
	GSC	FBS, CS/bFGF, SCF/mLIF	Suraeva et al. (2008)
	CEF	FBS, CS/bFGF, SCF, IGF-I/mLIF, IL-11	Shiue et al. (2009)
	Inactivated MEF	FBS, CS/bFGF, SCF, IGF-I/mLIF	J. Wang et al. (2009)
	CEF	FBS/bFGF/mLIF	Wu et al. (2010)

Abbreviations: BRL = buffalo rat liver cells, STO = Sandoz inbred mouse-derived thioguanine-resistant and ouabain-resistant fibroblast, CEF = chicken embryonic fibroblasts, GSC = gonadal stromal cells, MEF = mouse embryonic fibroblasts, FBS = Fetal bovine serum, CS = chicken serum, bFGF = basic fibroblast growth factor, SCF = stem cell factor, IGF-I = insulin growth factor type I, mLIF = murine leukaemia inhibitory factor, IL-11 = interleukin-11.
